# Therapeutic effect of natural polyphenols against glioblastoma

**DOI:** 10.3389/fcell.2022.1036809

**Published:** 2022-10-04

**Authors:** Ozal Beylerli, Aferin Beilerli, Alina Shumadalova, Xiaoxiong Wang, Mingchun Yang, Hanran Sun, Lei Teng

**Affiliations:** ^1^ Рeoples’ Friendship University of Russia (RUDN University), Moscow, Russia; ^2^ Department of Obstetrics and Gynecology, Tyumen State Medical University, Tyumen, Russia; ^3^ Department of General Chemistry, Bashkir State Medical University, Ufa, Russia; ^4^ Department of Neurosurgery, The First Affiliated Hospital of Harbin Medical University, Harbin, China

**Keywords:** glioblastoma, anticancer therapy, polyphenols, flavonoids, curcumin

## Abstract

Glioblastoma (GBM) is the most common and aggressive tumor of the central nervous system, which has a highly invasive growth pattern, which creates poor prospects for patient survival. Chemotherapy and tumor surgery are limited by anticancer drug resistance and tumor invasion. Evidence suggests that combinations of treatments may be more effective than single drugs alone. Natural polyphenolic compounds have potential as drugs for the treatment of glioblastoma and are considered as potential anticancer drugs. Although these beneficial effects are promising, the efficacy of natural polyphenolic compounds in GBM is limited by their bioavailability and blood-brain barrier permeability. Many of them have a significant effect on reducing the progression of glioblastoma through mechanisms such as reduced migration and cell invasion or chemosensitization. Various chemical formulations have been proposed to improve their pharmacological properties. This review summarizes natural polyphenolic compounds and their physiological effects in glioblastoma models by modulating signaling pathways involved in angiogenesis, apoptosis, chemoresistance, and cell invasion. Polyphenolic compounds are emerging as promising agents for combating the progression of glioblastoma. However, clinical trials are still needed to confirm the properties of these compounds *in vitro* and *in vivo*.

## Introduction

Glioblastoma (GBM) is the most common primary highly invasive glial tumor in the brain. It is characterized by proliferation of microvessels, pronounced necrosis, and resistance to modern methods of treatment (Erices et al., 2018; Huang et al., 2019; Shahcheraghi et al., 2019). GBM therapy includes radical removal of the brain tumor, radiation therapy and chemotherapy (Arevalo et al., 2017). The most popular chemotherapy drugs for gliomas are temozolomide (TMZ), lomustine, dacarbazine, procarbazine, vincristine, cisplatin, carmustine, nimustine, fotemustine, paclitaxel, and carboplatin (Schor, 2009; Mierzwa et al., 2010; Philip-Ephraim et al., 2012). The disadvantage of all these drugs is the limited ability to suppress interphase tumor cells. This standard of treatment has been used in practice for more than 20 years, but does not lead to a significant improvement in the condition of patients, but only increases the duration of the relapse-free period (Klinger and Mittal, 2016). Such treatment is non-radical, palliative, since the root cause of the disease is not solved. Currently, scientists have certain hopes for targeted chemotherapy drugs, antitumor immunotherapy, and pharmacogenomic technologies (Shah et al., 2013; Misso et al., 2014). When performing the standard protocol of complex treatment, the median survival of patients is 12–15 months, without treatment - 3 months. Despite all the efforts of doctors, only 25% of patients can survive 2 years from the moment of diagnosis, which is sad against the backdrop of significant progress in the treatment of other types of cancer and their localization (Gulati et al., 2012). This is most likely due to the fact that glial tumors are a very heterogeneous group of neoplasms both at the genetic and cellular levels. The pathogenesis of GBM is not well understood, but it has been proven that dysregulation of cell signaling pathways and genetic mutations play a crucial role in the onset, invasion, and progression of this disease (Shabaninejad et al., 2020). In addition, GBM is characterized by the absence of sharp boundaries between the tumor and healthy brain tissue, which makes it difficult to remove all tumor cells and causes tumor recurrence and growth (Sherriff et al., 2013). It is also worth noting the limited effectiveness of modern technologies in relation to tumor stem cells, the main reason for the resistance of malignant glial tumors. Thus, GBM is one of the most difficult problems of modern medicine and requires the development of new methods of treatment. In recent decades, the chemotherapeutic efficacy of natural polyphenolic compounds and their derivatives for the treatment of human malignant tumors has been actively studied (Shabaninejad et al., 2020).

Many natural polyphenolic compounds with proven biological efficacy have an oncogenic effect on glioblastomas *in vitro* and/or *in vivo*. These are flavonoids, curcuminoids, coumarins, alkaloids, carboxylic acid derivatives, carotenoids, terpenes, plant extracts, tannins and lignans, natural steroids. Among these compounds, curcuminoids and flavonoids, polyphenolic secondary plant metabolites, are of interest. Curcumin is a natural polyphenolic compound isolated from turmeric (Curcuma longa) and has enhanced therapeutic activity due to its neuroprotective, antioxidant, antiproliferative, and anti-inflammatory properties (Hesari et al., 2019). The antitumor potential of curcumin has generated a lot of interest due to its long-term human acceptance and reasonable safety. The antitumor activity of curcumin is due to the manifestation of biochemical mechanisms, including mutagenesis, oncogenesis, metastasis, cell cycle regulation, apoptosis, and autophagy (Kunati et al., 2018). It should be noted that the ability to penetrate the blood-brain barrier (BBB) and the lipophilic properties of curcumin make it a potential therapeutic and protective agent for malignant neoplasms of the central nervous system (Ahmed and Gilani, 2014).

Flavonoids have an antitumor effect through chemosensitization, metabolism modulation, inhibition of metastasis, and induction of apoptosis (Liskova et al., 2021; Samec et al., 2021). Based on these well-established oncostatic actions, flavonoids have great potential in modulating the response of GBM cells to anticancer drugs by overcoming their therapeutic resistance. The effectiveness of flavonoids in GBM is well documented in preclinical studies (Zhai et al., 2021). New therapeutic strategies will use evidence-based combinations of selected agents, each at a low dose, to create novel tumor cell-specific therapeutics. This review summarizes previous studies on the effectiveness of natural polyphenolic compounds, in particular curcuminoids, flavonoids, in the treatment of glioblastoma.

## Antitumor activity of flavonoids in glioblastomas

Flavonoids belong to the class of polyphenolic compounds of plant origin. They are classified as secondary products of plant metabolism. However, among secondary products, this group of substances is one of the most studied, which is associated with its participation in many key processes of plant development and growth (Gould et al., 2006). One of the most noticeable functions of flavonoids is their participation in the protection of plants from oxidative stress due to their pronounced antioxidant activity (Gould et al., 2006). The variety of flavonoids is huge and has about 8,000 substances. At the same time, it is known that up to 20% of the carbon fixed during photosynthesis goes to the production of polyphenolic compounds, among which flavonoids occupy a significant place (Harborne and Williams, 2000; Ververidis et al., 2007). In animal and human cells, flavonoids are not synthesized, and the presence of flavonoids in tissues depends entirely on the consumption of plant products (Mennen et al., 2008). Bioactive flavonoids are united by a three-ring structural backbone, including two phenyl rings and one central heterocyclic ring. These compounds are classified on the basis of structural differences associated primarily with the presence and location of substituents in the heterocycle. Interest in flavonoids is due not only to the possible positive effects of these substances observed when using plant products, but also to the prospect of obtaining synthetic derivatives of these substances with a therapeutic effect. Based on flavonoids, it is possible to create new highly active drugs with anticarcinogenic, anti-inflammatory, antiviral, bactericidal or antiparasitic activity. Based on flavonoids, new antibiotics are being created and tested, as well as agents that enhance the effect of other drugs due to the ability of flavonoids to suppress the mechanisms of multiple drug resistance. The main flavonoids and their effects are shown in Table 1.

**TABLE 1:** Influence of oral intake of flavonoids and flavonoid-containing plant products on intracellular signaling systems of the brain and cognitive functions.

Flavonoid	Mechanism of action
Cocoa decoction, cocoa epicatechin	Improving memory and learning, reducing the risk of Alzheimer’s disease and stroke, increasing the viability of neurons during intoxication, increasing synaptic plasticity
Flavonoid extracts from ginkgo leaves (Ginkgobiloba)	Increased levels of extracellular dopamine and acetylcholine
Juices or flavonoids from blueberries and strawberries (strawberries), blackberries, grapes, plums	Reducing the risk of cognitive decline in the elderly, a positive effect on the cognitive functions of rodents, increasing the activity of microglia. Activation of NF-kB and MAPK
Green tea EGCG or green tea decoction	Neuroprotective activity, improvement of cognitive functions, improvement of attention, tranquilization and anxiolytic action, action on the cholinergic system, glutathione system, CREB and Bcl-2 systems, protection against oxidative stress
Grape proanthocyanidins	Improved memory, synaptic plasticity, learning ability, reduced risk of Alzheimer’s disease
Acai palm anthocyanidins	Protective effect on microglial cells, reduction of COX-2, p38, TNF-α, NF-kB
Red yam polyphenols	Improvement of cognitive functions, enhancement of biogenesis of mitochondria of hippocampal neurons
Naringenin, Naringin	Tranquilizing and anxiolytic action, improvement of immobilization stress tolerance, neuroprotective, anti-inflammatory, antioxidant action, interaction with the binding site of diazepine, GABA receptor, protection of mitochondria, increase in the level of TNF-α in the brain
Pycnogenol from Maritime Pine	A study on students found improvements in attention, memory, performance, and mood. Relief of menopausal symptoms in older women
Alcoholic extract of morinda citrus fruit (Morinda citrifolia L.)	Improving memory, increasing cerebral blood flow, inhibiting oxidative stress and acetylcholinesterase activity
Genistein	Improving memory and learning, long-term improvement in cognitive functions in Sanfilippo disease
Silymarin	Protection against oxidative stress, Mn chelation, acetylcholinesterase activation, improvement in Alzheimer’s disease
Silibinin	Improvement of memory, reduction of oxidative stress in the brain of diabetic mice, effect on the cholinergic system, improvement of brain energy metabolism, inhibition of beta-amyloid aggregation
Extra virgin olive oil	Improving memory and learning in older mice, reducing the manifestations of Alzheimer’s disease
Walnuts	After 8 weeks of consumption, college students improved their verbal logic test scores by 11.2%. Changes in the non-verbal test in logic, memory and mood were not found
7,8-dihydroxyflavone	Tyrosine kinase B (TrkB) receptor agonist involved in the pathogenesis of Alzheimer’s. The molecule passes through the blood-brain barrier
Luteolin	Antidepressant. At a concentration of 1–10 μM, it prevents neuronal death and affects the expression of stress proteins in the hippocampus
Liquritigenin	Improvement of memory and learning ability, inhibition of hippocampal astrocytes and Notch-2 signaling pathway related to Alzheimer’s disease
2′-methoxy-6-methylflavone	Sedative and anxiolytic action. GABA(A) receptor activator and modulator
Maureen	Therapy for Alzheimer’s disease, reduction of Ʈ-protein phosphorylation and filament tangle formation in the hippocampus
Quercetin, Rutin	Improvement of memory and learning ability in animals after intoxication, protection of hippocampal neurons
Hesperidin	Anxiolytic action, memory improvement after intoxication
Glabridin	Memory and learning retention in diabetic rats
Soy isoflavones	Improving memory and learning abilities in animal experiments (in humans, data are inconsistent). Protection against inflammation induced by beta-amyloid in Alzheimer’s disease, suppression of the expression of NF-kB and Toll-like receptor, increased energy of brain tissue mitochondria
Baicalein	Sedative and anxiolytic effects, action on the GABA system
Apigenin	Protecting the brain from the toxic effects of beta-amyloid
Troxerutin	Protection of the mouse brain from high cholesterol, manifestations of diabetes and Alzheimer’s disease, protection of neurons from apoptosis
Icariin	Neuroprotective action against oxidative stress and neurodegeneration, MAPK activation, neuronal protection in Alzheimer’s disease in mice, curative action in patients with mild cognitive impairment
Abakopterin E from fern (Abacopteris penangiana)	Protecting neurons from oxidative stress, improving memory and learning in animals
Daidzein, Daidzin	Normalization of cognitive functions in animals with disorders of the cholinergic system
Fisetin	Neuroprotective effect in animals with Huntington’s disease, effect on protein kinases of the ERK cascade
Nobiletin from citrus fruits	Memory enhancement, antidepressant, action on the noradrenergic and dopamine systems

Most of the experiments were carried out *in vivo*.

Many studies conducted over the past decades, including *in vitro* experiments using various tumor cell cultures, *ex vivo* experiments on animal models, as well as a number of epidemiological studies, have shown that flavonoids are able to inhibit the three stages of cancer cell development: initiation, activation and distribution. In the early stages, this effect includes an effect on oxidative stress, inactivation of carcinogens, and inhibition of cell proliferation. At the reproduction stage, the effectiveness of flavonoids most likely lies in the inhibition of angiogenesis, the suppression of tumor metastasis, and the activation of apoptosis (Middleton et al., 2000; Mantena et al., 2005; Chachar et al., 2011; Kilani-Jaziri et al., 2012; Majewski et al., 2012; Li et al., 2013a; Li et al., 2013b; Li et al., 2014).

The main role in the mechanism of antitumor action of flavonoids is played by their ability to inhibit the proliferation of malignant cells. This is largely due to the inhibitory effect of these polyphenols on the chain of biochemical events associated with cell growth (Kandaswami et al., 2005). For example, early studies have shown that quercetin inhibits aerobic glycolysis in tumor cells and also inhibits protein synthesis in cell cultures of a number of tumors and increases the level of cyclic adenosine monophosphate (AMP) in these cells (Graziani et al., 1977; Jullien et al., 1984). In this context, we note the role of 5′AMP-activated protein kinase AMPK (AMPK), which controls the energy balance of the cell. By blocking the synthesis of fatty acids and accelerating their oxidation, AMPK regulates the cell cycle and cell proliferation. Activation of AMPK primarily inhibits the development of cancer cells, which is combined with the stimulation of their apoptosis. Therefore, it is not surprising that the use of chrysin flavone, by activating AMPK, inhibited the growth of lung cancer cells and induced their apoptosis (Shao et al., 2012). It has recently been suggested that the antiproliferative effect of the flavone hesperetin on breast cancer cells is due to the suppression of glucose uptake (Yang et al., 2013). A similar effect was later found in the flavonol kaempferol (Azevedo et al., 2015). Recently, information has appeared on the important role of the Wnt signaling pathway involved in the control of cell differentiation, proliferation, and cell motility. Cell mobility is acquired during the epithelial-mesenchymal transition and is necessary for invasion and metastasis. Dysregulation, mutational changes in this pathway contribute to the development of glioblastoma (Amado et al., 2011). Evidence has emerged that the antiproliferative activity of flavonoids may be related to their ability to inhibit this pathway at various levels. At least isoquercetin, genistein, isorhamnetin, a glycosylated form of quercetin, and epigallocatechin gallate (EGCG) directly inhibited nuclear translocation of the β-catenin protein (an important modulator of the Wnt pathway), exerting a significant antiproliferative effect against glioblastoma tumor cells (Orfali et al., 2016).

A large number of studies on the antitumor effect of flavonoids concerns their effect on apoptosis. It is known that the activation of apoptosis is one of the most important ways to suppress the growth of cancer cells by anticancer drugs. Apoptosis is a process of cell suicide involving an extrinsic pathway associated with the death receptor and a mitochondria-dependent intrinsic pathway involved in tissue development and homeostasis in multicellular organisms. The receptor-dependent pathway is provided by activation by ligands and transmembrane death receptors, which, through stimulation of intracellular adapter proteins, cause the activation of initiator caspases that form the DISC signaling complex. The mitochondrial pathway is realized through the action of such proapoptotic factors as Bak, Bax, Bak/Mtd. These factors increase the permeability of the outer mitochondrial membranes, ensuring the release of procaspase, soluble cytochrome C proteins, and AIF from the intermembrane space into the cytoplasm. Both pathways, which usually intersect at some point, lead to the activation of effector initiation caspases, leading to the formation of apoptotic bodies. An important role in the regulation of apoptosis, in addition to the proapoptotic factors mentioned above, is played by the antiapoptotic factors Bcl-XL, Bcl-2, Bcl-w, *etc.*, as well as the p53 transcription factor, the most well-known tumor suppressor that acts as a DNA damage sensor. Studies of recent decades convincingly show that a number of dietary flavonoids are able to induce apoptosis in various models of carcinogenesis (Surh, 2008; Amin et al., 2009; Pratheeshkumar et al., 2012). At the same time, the anticancer activity of EGCG, luteolin, quercetin, daidzein, genistein, and apigenin, discovered in recent years, is associated with the induction of apoptosis (Hu, 2011; Kim et al., 2012; Pan et al., 2012). It should be noted that the stimulation of apoptosis contributes to the solution of one of the key problems in the treatment of malignant tumors - the resistance of tumor cells to the action of cytostatics. And in this regard, flavonoids, many of which activate apoptosis, can make a significant contribution to the conservative treatment of malignant neoplasms. Unfortunately, despite the fact that our knowledge of the signaling pathways of apoptosis has expanded significantly in recent years, much of the mechanism of action of flavonoids remains not fully understood.

Tumor angiogenesis is one of the factors that increase the malignant potential of the tumor and promotes metastasis, despite a certain inferiority of the newly formed vessels. At the same time, a number of factors such as vascular endothelial growth factor (VEGF), hypoxia, fibroblast growth factors (FGF-1, FGF-2), platelet growth factor (PDGF), angiopoietin-1 (ang-1) and others, produced by the stroma, endothelium, tumor cells and blood, as well as the extracellular matrix, are able to stimulate tumor angiogenesis. Accordingly, there are also factors in the form of anti-angiogenic molecules that prevent the formation of tumor vessels. The predominance of angiogenic factors over antiangiogenic factors largely determines the proliferation of the tumor and its metastasis. In this case, one of the key moments of angiogenesis is the destruction of the basement membrane, which causes the migration of endothelial cells, which is necessary for the process of neovascularization (Sounni et al., 2011; Basagiannis et al., 2016). As it turned out, the antitumor activity of a number of flavonoids may be due to their inhibitory effect on neoangiogenesis. An anti-angiogenic potential, for example, has been found in EGCG (Singh et al., 2002). The flavone apigenin suppressed tumor angiogenesis in both *in vitro* and *in vivo* experiments by reducing the expression of VEGF and hypoxia-inducing factor HIF-1α. In another study, apigenin inhibited the expression of VEGF and erythropoietin mRNA, which is a typical hypoxia-induced gene, through the degradation of HIF-1α (Osada et al., 2004). In addition, it was shown that apigenin significantly inhibited VEGF/FGF-induced stimulation of the activity of MMP-1 and MT1-MMP metalloproteinases and the activity of the plasminogen activator PAI-1, and also ensured the activation of MMP inhibitors, which together provided inhibition of angiogenesis (Kim, 2003; Deep and Agarwal, 2010; Kelly, 2011; Singh et al., 2011; Tan et al., 2016; Ganeshpurkar and Saluja, 2017; Patel and Patel, 2017; Mani and Natesan, 2018; Tay et al., 2019).

Various flavonoids, including flavan-3-ols, flavones, isoflavones, flavonols, flavonol glycosides, and flavonolignans, are effective against GBM when combined with chemotherapy drugs *in vitro* and/or *in vivo* (Table 2).

**TABLE 2:** Major classes of flavonoids interacting with chemotherapeutic agents to inhibit GBM.

Source	Class	Flavonoid	Ref
Milk thistle (Silybum marianum)	Flavonolignan	Silibinin	Deep and Agarwal, (2010)
Rue (Ruta graveolens)	Flavonol Glycoside	Rutin	Ganeshpurkar and Saluja, (2017)
Horny goat weed (Epimedium)	Flavonol Glycoside	Icariin	Tan et al. (2016)
Oak (Quercus)	Flavonol	Quercetin	Kelly, (2011)
Red clover (Trifolium pratense)	Isoflavone	Formononetin	Tay et al. (2019)
Gumweed (Grindelia argentina)	Flavone	Hispidulin	Patel and Patel, (2017)
Passionflower (Passiflora)	Flavone	Chrysin	Mani and Natesan, (2018)
Green and white tea	Flavan-3-ol	epigallocatechin-3-gallate (EGCG)	Singh et al. (2011)

One of these drugs, arsenic trioxide (ATO), has a pleiotropic antitumor effect due to the formation of ROS and regulation of the cell cycle (Hoonjan et al., 2018). In glioma cells, ATO induces caspase-independent autophagic cell death (Kanzawa et al., 2005). Moreover, combinations of ATO and TMZ, ATO and vismodegib show a synergistic effect on the growth of GBM *in vivo* (Bureta et al., 2019). Chloroquine, another compound of interest, is a repurposed antimalarial drug that induces p53-dependent apoptosis and disrupts the mitochondrial membrane potential in glioma cells (Kim et al., 2010). A recent clinical study, in combination with a standard regimen of radiation and chemotherapy, examined its efficacy against GBM (Sotelo et al., 2006). As a widely used chemotherapeutic agent, cisplatin, a platinum-based DNA alkylating agent, has been clinically tested in various cancers, including GBM. Mechanically, the effects of cisplatin against GBM are due to p53-dependent apoptosis (Park et al., 2006). Similarly, the natural topoisomerase II inhibitor etoposide has undergone extensive clinical trials in GBM. Etoposide induces apoptosis of glioma cells by successively generating ceramide, modulating Bax/Bcl-2, releasing cytochrome C, and activating caspase (Sawada et al., 2000). Finally, sodium butyrate is a short-chain fatty acid histone deacetylase inhibitor that reduces glioma cell cell cycle progression, proliferation, and migration (Engelhard et al., 2001). Although sodium butyrate has potential against GBM, its effects are not currently confirmed by clinical trials.

## Antitumor activity of curcumin in glioblastomas

Curcuma (Curcuma longa), a plant belonging to the ginger family (Zingiberaceae), has long been recognized for its medicinal properties, and curcumin has been found in its rhizome (Amalraj et al., 2016; Tsuda, 2018; Kotha and Luthria, 2019). It is claimed that turmeric has been widely used in the treatment of various diseases for over 2500 years, mainly in Asian countries (Deogade and Ghate, 2015; Kocaadam and Sanlier, 2015). Current evidence suggests that curcumin may be useful in the treatment of a wide range of human diseases, including Alzheimer’s disease (Lopresti, 2017), Parkinson’s disease, diabetes, cardiovascular disease, arthritis, and various neoplasms, including brain tumors (Klinger and Mittal, 2016; Shabaninejad et al., 2020). Curcuma contains approximately 70% carbohydrates, 13% moisture, 6% protein, 6% essential oils, 5% fat, 3% minerals, 3–5% curcuminoids, and trace amounts of vitamins (Prasad et al., 2014; Nelson et al., 2017). Curcuminoids, in turn, consist of curcumin (77%), dimethoxycurcumin (17%), and bisdimethoxycurcumin (3%) (Chen et al., 2017). It should be noted that other curcuminoids and their synthetic derivatives also exhibit biological activity (Luthra and Lal, 2016). Curcumin is a polyphenolic compound that makes up 2–5% of turmeric powder (Ahmed and Gilani, 2014; Kunati et al., 2018). Curcumin is able to cross the BBB and accumulate in the hippocampus. The presence of a large amount of lipids in the brain, the lipophilic properties of curcumin contribute to its availability and assimilation by the cells of the central nervous system (Shabaninejad et al., 2020). The results of preclinical and clinical studies have demonstrated the chemotherapeutic potential of curcumin in various types of neoplastic diseases, such as lymphomas, multiple myeloma, melanoma, cancer of the skin, lung, prostate, breast, ovary, bladder, liver, gastrointestinal tract and pancreas (Luthra and Lal, 2016; Hosseini and Hosseinzadeh, 2018). The therapeutic effects of curcumin have been studied in numerous studies, and today a wide range of its pharmacological activities is presented. Curcumin has antimicrobial, anti-inflammatory, antiatherosclerotic, antioxidant, antiangiogenic, and antitumor activity (Luo et al., 2019). Curcumin affects carcinogenic signaling pathways associated with cell proliferation, apoptosis, autophagy, angiogenesis, immune response, invasion and metastasis through transcription factors, receptors, kinases, cytokines, enzymes and factors growth (Zanotto-Filho et al., 2015; Carolina Alves et al., 2019; Mortezaee et al., 2019). It should be noted that curcumin reduces the proliferation of GBM cells and their radio- and chemo-resistance. The molecular mechanisms of this action are associated with the transcription factors AP-1 and NF-κB and are due to the inhibition of JNK and AKT expression (Dhandapani et al., 2007; Kunati et al., 2018). Decreased expression of Bcl-2 and DNA repair enzymes such as MGMT, ERCC-1, DNA-PK, Ku70 and Ku80 results in resistance of glioma cells to chemotherapy and radiotherapy, but these cells remain sensitive to the action of curcumin. This feature suggests that the combination of curcumin with chemotherapy drugs or radiation therapy may increase the sensitivity of tumor cells to chemotherapy or radiation therapy (Mortezaee et al., 2019; Trotta et al., 2019).

Malignant glioma cells are extremely invasive. They easily migrate and penetrate into the surrounding brain parenchyma, leading to the progression of the disease (Park et al., 2019). One of the methods by which GBM tumor cells are able to penetrate into normal brain tissue is the abnormal expression of matrix metalloproteinases (MMPs), membrane-associated or secreted enzymes involved in the degradation of the extracellular matrix (Klinger and Mittal, 2016; Luthra and Lal, 2016). MMP-1 and MMP-3 are associated with glioma invasiveness, MMP-2 and MMP-9 play a major role in invasion and migration, and MMP-9 is involved in angiogenesis (Ghosh et al., 2015; Luthra and Lal, 2016). It was previously shown that curcumin suppresses the expression of MMP-1, -3, -9 and -14 in MGB cell lines U87MG and U373MG by inhibiting AP-1 and MAP (Klinger and Mittal, 2016; Luthra and Lal, 2016). Inhibition of the MMP-9 level in U87MG cells was carried out by suppressing the expression of MAPK (p38, JNK, ERK) (Luthra and Lal, 2016). Urokinase-type plasminogen activator (uPA) is a serine protease that triggers a degradation cascade by converting extracellular plasminogen to plasmin, then degrading extracellular matrix collagen and activating other MMPs. Curcumin is able to reduce uPA activity by preventing RelA/NF-κB translocation to the cell nucleus (Klinger and Mittal, 2016). Meng et al. showed that curcumin suppressed the activity of the Hedgehog signaling cascade by affecting the expression of Gli1, SMO, and Sufu proteins, as well as inhibiting the epithelial-mesenchymal transition in various tumor cells (Meng et al., 2017). It is suggested that fascin is overexpressed in GBM cells and may be associated with the migration and invasion of glioma cells. The results of recent studies have shown that curcumin negatively regulates the expression of fascin in GBM cells by influencing the STAT3 signaling cascade (Park et al., 2019). An important factor involved in invasion and metastasis of tumors is angiogenesis (Klinger and Mittal, 2016). The interaction between vascular endothelial growth factor (VEGF) and platelet growth factor (PDGF) with rapamycin (mTOR) in mammals activates angiogenesis (Saberi-Karimian et al., 2019). Curcumin affects the entire process of angiogenesis by suppressing the expression of transcription factors such as NF-κB and proangiogenesis factors VEGF, bFGF and MMPs (Wang and Chen, 2019). bFGF induces angiogenesis by acting on smooth muscle cells and endothelial cells, as well as acting as a chemoattractant, and promotes the proliferation of fibroblasts and epithelial cells (Wang and Chen, 2019). MMP-9, in turn, increases the efficiency of VEGF responsible for proliferation, BBB permeability, and angiogenesis (Wang et al., 2017). Most of the biological properties of VEGF are due to its high affinity for the VEGFR-1 and VEGFR-2 receptors on vascular endothelial cells, as well as for the VEGFR-3 receptor (Saberi-Karimian et al., 2019; Wang and Chen, 2019). Results obtained in a mouse model suggest that curcumin may inhibit VEGF/Ang-2/TSP-1 mediated angiogenesis and tumor growth (Luo et al., 2019).

**FIGURE 1:**
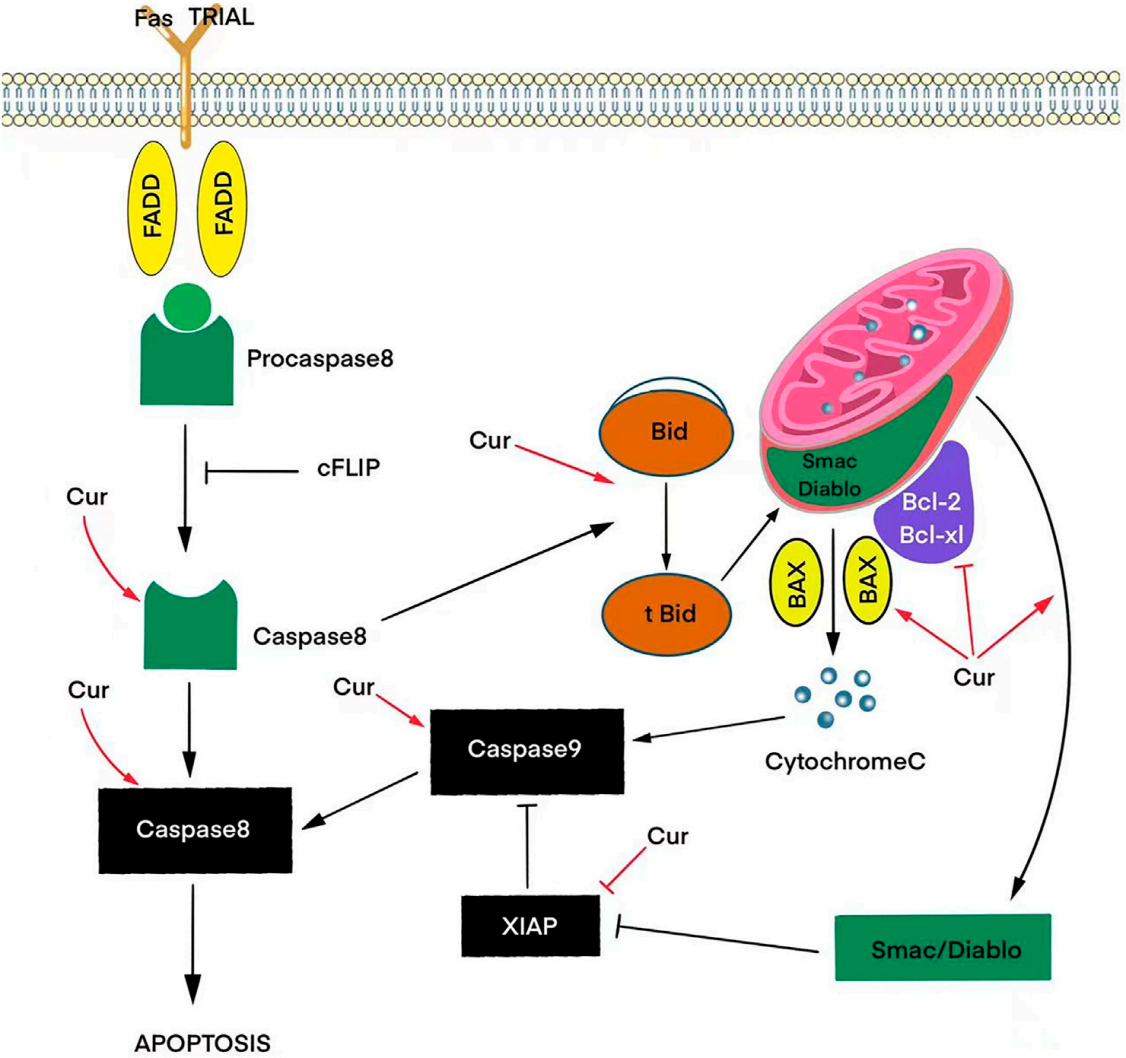
Effects of curcumin (Cur) on signaling pathways that trigger apoptosis *via* activation of pro-apoptotic proteins (BAX) and inhibition of anti-apoptotic proteins (Bcl-2, Bcl-xL, XIAP) to release the caspase and cytochrome C cascade.

Apoptosis, as a complex programmed process, includes receptor-dependent (external) and mitochondrial (internal) signaling pathways. Curcumin-mediated induction of the extrinsic pathway depends on Fas/CD95/TRAIL signaling activating caspases 8 and 10. The effects of these caspases converge in the intrinsic pathway, generating a mitochondrial potential, causing Bid cleavage and cytochrome C release. Cytochrome C in turn activates the proteins of the family Bcl-2, which triggers a cascade of caspases leading to cell death in response to cellular signals, including stress or DNA damage. Also, together with sitochrome C, the proapoptotic molecule Smac/Diablo is released from mitochondria in curcumin-induced T98G and U87MG cells, which suppresses IAPs in the cytosol, which facilitates the process of apoptosis (Figure 1) (Luthra and Lal, 2016).

In DBTRG glioma cells, the action of curcumin increases the expression of Bax and caspase 3 and reduces the expression level of Bcl-2, inducing apoptosis through an intrinsic pathway. Curcumin also actively influences CDKN2A/p16 by suppressing phosphorylated RB (Su et al., 2010). The results of a study using the 8401 GBM cell line showed that curcumin reduces cell proliferation, reduces the potential of the mitochondrial membrane, induces DNA fragmentation, induces apoptosis through a caspase-dependent pathway (caspase 3, 8, and 9), and inhibits the activity of the transcription factor NF-κB (Dhandapani et al., 2007). In the U87MG cell line, curcumin can induce apoptosis by suppressing anti-apoptotic signals, stimulating caspase 8 activation, and increasing the BAX/Bcl-2 ratio. The authors of this study also reported that the expression of cyclin D1, NF-κB, AKT, ERK, and Bcl-xL is suppressed when B16F10 cells are treated with curcumin (Klinger and Mittal, 2016).

The use of TMZ in adjuvant chemotherapy of GBM leads only to a slight increase in the median survival of patients (up to 14.6 months), mainly due to the formation of multidrug resistance that affects the activity of O-(6)-methylguanine-DNA-methyltransferase. Curcumin in combination with TMZ seems to have an additive cytotoxic effect on GBM cells (Zanotto-Filho et al., 2015). In addition, both drugs cause cell cycle arrest in the G2/M phase through the activation of proteins such as Wee, Cdc2, CHK1, and Cdc25c (Zanotto-Filho et al., 2015). Treatment with TMZ with curcumin has been shown to induce autophagy, which is ERK1/2 dependent and is associated with inhibition of STAT3, NF-κB, and PI3K/AKT (Klinger and Mittal, 2016). Yin et al. found a synergistic effect of the combination of curcumin and TMZ in the generation of reactive oxygen species (ROS) (Yin et al., 2014). Gersey et al. suggested that curcumin targets GBM stem cells through ROS induction, possibly by decreasing STAT3 activity (Gersey et al., 2017). In addition, curcumin enhances the action of paclitaxel, cisplatin, etoposide, camptothecin, and doxorubicin in T98G, U87MG, and U138MG cells (Luthra and Lal, 2016). The LN18 and U138MG cell lines treated with 20 μM curcumin and 10 nM paclitaxel were found to have a combination index of 0.1 and 0.09, respectively, indicating a synergistic effect. This combination activated caspase 3, caspase 8, and calpain and increased the BAX/Bcl-2 ratio. In addition to the synergistic effect, curcumin may prevent chemoresistance (Hossain et al., 2012; Soleimani et al., 2018). The results of a study conducted in 2008 on Sprague-Dawley rats showed that curcumin suppressed the expression of a protein associated with multidrug resistance, ABCG2 (Klinger and Mittal, 2016; Chen et al., 2017). Three curcuminoids monomers reduced the expression of ABC transporters, including ABCB1, ABCG2, and ABCC1, in tumor cells without causing systemic toxicity (Chen et al., 2017). Curcumin differently sensitized apoptosis in U251MG and U87MG cells that were resistant to TRAIL therapy. The study of the mechanism of the combined effect of curcumin and TRAIL therapy on U87MG cells showed that the cleavage of procaspases 8, 9, 3 and cytochrome C released from mitochondria leads to apoptosis. It was also found that the biological activity of curcumin is associated with the presence of hydroxyl and methoxy groups, which contributed to the development and synthesis of new analogs of curcumin.

Cell cycle arrest in any of the four phases leads to inhibition of cell proliferation and survival (Trotta et al., 2019). There is ample evidence suggesting that curcumin is involved in the modulation of most GBM signaling pathways in both the cytosol and the nucleus (Klinger and Mittal, 2016). Molecular interactions of curcumin include inhibition of proliferation and reduced survival of glioma cells through effects on key signaling cascades: PI3K/AKT, RAS, and JAK/STAT, including EGFR/PI3K/PTEN/RAS/STAT3; cell cycle modulation pathway, including changes in TP53/MDM2/MDM4/p14ARF and RB1/CDK4/p16INK4A/CDKN2B (Ghosh et al., 2015; Luthra and Lal, 2016). Overexpression of EGFR (epidermal growth factor receptor) induces downstream signaling pathways such as PI3K/AKT, RAF/MEK/ERK and JAK/STAT, but the mechanism of attenuated ERK signaling has shown that it may not necessarily lead to increased downstream signaling (Trotta et al., 2019). DBRTG glioma cells treated with curcumin did not alter the level of EGFR and the activity of its associated downstream pathways (RTK/RAS/PI3K) in the cytosol. In U87MG and U138MG cells, proliferation was inhibited by EGFR action on ERK and the downstream PI3K signaling cascade, respectively (Luthra and Lal, 2016). Curcumin inhibited phosphorylation of AKT/PKB and its downstream substrate p70S6K, NF-κB along with its regulated cytoprotection genes (IAPs and bcl family members) and DNA repair enzymes (MGMT, Ku70, Ku80 and DNA-PKcs) in U87MG cells, resulting in inhibition of cell proliferation and survival (Trotta et al., 2019). The results of a number of studies have shown that DAPK1 plays an important role in curcumin-induced cell death (Klinger and Mittal, 2016). DAPK1 mediated the antiproliferative and proapoptotic effects of curcumin through the regulation of STAT3 and NF-κB signaling pathways and inhibition of caspase 3 (Trotta et al., 2019). The results of other studies on U251MG MGB cells demonstrated that curcumin increased p53 expression, causing cell cycle arrest in the G2/M phase and increasing the expression of the tumor suppressor ING4 (Trotta et al., 2019). Along with triggering apoptosis, p53 also inhibits cell cycle and cell senescence in malignant tumors (Mortezaee et al., 2019). In DBRTG glioma cells, curcumin increased p53 protein levels and also inhibited the RB signaling pathway, B1/CDK4/p16INK4, involved in cell cycle activation in the G1/S phase *via* upregulation of CDKN2A/p16 and downregulation of phosphorylated RB, arresting cells in the G1 phase/S (Trotta et al., 2019). It is believed that curcumin has an anti-inflammatory effect due to the hydroxyl and methoxy groups (Deogade and Ghate, 2015). Curcumin down-regulates pro-inflammatory interleukins (IL-1, -2, -6, -8 and -12), cytokines (TNFα, monocytic chemoattractant protein 1), thereby suppressing the JAK/STAT signaling pathway. It is also claimed that curcumin regulates the inflammatory response by suppressing the activity of the enzymes inducible nitric oxide synthase (iNOS), cyclooxygenase 2, lipoxygenase, and xanthine oxidase, which may lead to suppression of NF-κB activation (Kocaadam and Sanlier, 2015). It is noted that the biological effects of curcumin appear to be largely dependent on the administered dose. Thus, low doses, in the range from 25 to 50 mg/kg, caused significant immunostimulatory effects *in vivo*, while higher concentrations led to immunosuppressive effects due to a decrease in the proliferation of various immune cells (Schnekenburger et al., 2019).

## Conclusion

Summing up the review, we note that today there is no doubt about the beneficial effect of natural polyphenolic compounds on the human body, due to their high biological activity. The antitumor properties of natural polyphenolic compounds found in spices and herbs have long attracted the attention of oncologists. In the treatment of GBM, various clinical methods are used, including surgery, radiation therapy and chemotherapy, but so far the standard therapy for GBM does not lead to satisfactory results. In this regard, new more effective therapeutic approaches and drugs are needed, taking into account the aggressive, diffuse nature and chemo- and radioresistance of the tumor.

In recent decades, it has been established that the biological activity of flavonoids is by no means exhausted by the above types of action. In addition to the well-known antioxidant, anti-inflammatory and antitumor effects, such activities as anti-ischemic, antihypertensive, antidiabetic, antimicrobial, antiviral, antithrombogenic, estrogenic, neurotropic, *etc.* should be noted. This is indirectly confirmed by a huge number of epidemiological studies conducted in recent years. At the same time, there are many problems that prevent both the targeted clinical use of flavonoids and the creation of individual highly effective drugs based on them. The first of them is determined by the features of the pharmacokinetics of flavonoids. The vast majority of the identified types of pharmacological activity has been confirmed in *in vitro* experiments, but it is far from always possible to achieve their adequate concentration in the body due to the characteristics of metabolism. However, most clinicians are justifiably wary of a significant increase in dosage due to possible and not yet established side effects. In addition, the mechanisms of their pharmacological action, taking into account modern approaches to the requirements of evidence-based medicine, need further in-depth comprehensive study. Nevertheless, we are close to an optimistic view of the prospects for the clinical use of flavonoids, which, in addition to the revealed diversity of biological activity, is due to the relative cheapness of obtaining drugs and the high prevalence of these food polyphenols in the nature around us.

Curcumin-based multitarget chemotherapy treatment may become the most relevant for GBM. This polyphenolic compound has low toxicity and has a number of pleiotropic properties, including anti-inflammatory, antioxidant and antitumor effects. The synergistic effects of curcumin with radiotherapy and chemotherapy have shown its potential for treating GBM. Moreover, curcumin-induced multimolecular targeting of various signaling pathways involved in cancer development may make curcumin one of the potential leaders in modern antitumor chemotherapy. However, despite the antitumor activity of curcumin in preclinical models and the good tolerance of high doses of the drug, its low bioavailability is still a problem that needs to be addressed. Other structural analogs of curcumin may be more bioavailable and potent, and designed to fit better in large, well-controlled clinical trials. Multipurpose studies are needed to determine the pharmacological efficacy of curcumin, its analogs and metabolites.
